# Human acute kidney injury is associated with a proinflammatory phenotype

**DOI:** 10.1186/cc13565

**Published:** 2014-03-17

**Authors:** N Gauge, M Varrier, D Boardman, M Hernandez-Fuentes, M Ostermann

**Affiliations:** 1 Guy's St Thomas' Hospital, London, UK

## Introduction

Animal research suggests that acute kidney injury (AKI) is an inflammatory condition [1]. Our aim was to describe the immune phenotype in human AKI.

## Methods

We enrolled patients with: AKI grade II/III (defined by KDIGO criteria) and systemic inflammatory response syndrome (SIRS) without sepsis; SIRS without AKI; and AKI II/III without SIRS. A healthy control population was used for baseline comparison. Serial blood samples were taken on days 0, 2 and 7. Cells were separated using Percoll gradients and phenotyped using flow cytometry.

## Results

The results from 24 day 0 samples identified statistically significant differences between SIRS, AKI, AKI + SIRS and healthy controls amongst: CD8+ cytotoxic T cells, CD45CD25^+++ ^regulatory T cells, and CD45CD25^++ ^cytokine secreting non-T-regulatory cells (Table 1). The percentage of CD69-positive neutrophils was significantly increased across all three groups relative to controls, with little variation between AKI, SIRS and AKI + SIRS patients.

**Table 1:** 

	AKI + SIRS	AKI no SIRS	SIRS alone	Control
% CD8^+ ^cytotoxic T cells	23.3	16.7	11.6	31.1
% Fr. II T-regulatory cells	4.1	2.7	1.6	1.2
% Fr. III cytokine T cell	11.5	21.2	13.9	8.0
% CD69^+ ^neutrophils	83.9	69.7	65.5	7.35

## Conclusion

Human AKI is associated with a proinflammatory phenotype.
